# Cetuximab enhances radiosensitivity of esophageal squamous cell carcinoma cells by G2/M cycle arrest and DNA repair delay through inhibiting p‐EGFR and p‐ERK

**DOI:** 10.1111/1759-7714.14995

**Published:** 2023-06-20

**Authors:** Guifang Zhao, Liwen Feng, Ting Ye, Yanshen Liu, Li Fan, Chengzhi Ye, Jing Chen

**Affiliations:** ^1^ Cancer Center, Union Hospital, Tongji Medical College Huazhong University of Science and Technology Wuhan China; ^2^ Department of Oncology, Jiangxi Provincial People's Hospital The first affiliated Hospital of Nanchang Medical College Nanchang China; ^3^ Department of Pediatrics Renmin Hospital of Wuhan University Wuhan China

**Keywords:** cetuximab, DNA‐PK, EGFR, radiation resistance, γH2AX

## Abstract

**Background:**

Although radiotherapy has improved local control in esophageal squamous cell carcinoma (ESCC), a considerable number of patients still experience relapse due to resistance. In this study, we aimed to evaluate the effects of cetuximab on radiosensitivity in two ESCC cell lines (ECA109 and TE‐13) and to investigate their underlying mechanisms.

**Methods:**

Cells were pretreated with or without cetuximab before irradiation. The MTT assay and clonogenic survival assay were performed to evaluate cell viability and radiosensitivity. Flow cytometry was performed to analyze cell cycle distribution and apoptosis. The γH2AX foci were counted to determine cellular DNA‐repairing capacity via immunofluorescence assay. The phosphorylation of key molecules involved in the epidermal growth factor receptor (EGFR) signaling pathway and DNA double‐strand break (DSB) repair were measured by western blot.

**Results:**

Cetuximab alone was not sufficient to suppress cell viability, but significantly enhanced radiation‐induced inhibition of clonogenic survival in ECA109 and TE‐13. The radiation sensitivity enhancement ratio (SER) was 1.341 and 1.237 for ECA109 and TE‐13, respectively. ESCC cells treated with cetuximab were arrested at the G2/M phase in response to radiation. No significant increase in apoptotic rate was observed in irradiated cells that were treated with cetuximab. The average number of γH2AX foci increased in the combination group (cetuximab and radiation). Cetuximab suppressed phosphorylation of EGFR and downstream ERK, but had no significant effect on AKT.

**Conclusions:**

These results indicate the potential for use of cetuximab as an effective radiosensitizer in ESCC. Cetuximab promotes G2/M cycle arrest and reduces DSB repair, as well as inhibiting EGFR and downstream ERK pathways in ESCC.

## Graphical abstract

Cetuximab improves radiosensitivity in ESCC cell lines ECA109 and TE‐13. It may act by promoting G2/M cycle arrest and reducing DSB repair, as well as inhibiting EGFR and downstream ERK pathways.

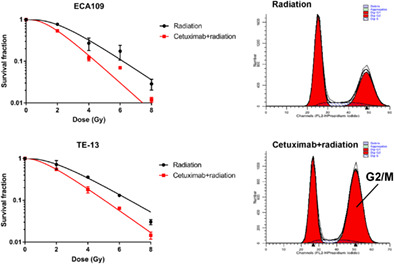

## INTRODUCTION

Esophageal cancer (EC) is considered a highly aggressive malignancy and is associated with a poor prognosis. Although EC ranks seventh among cancers worldwide with an incidence of 604 000, it ranks sixth in mortality, 544 000 annually worldwide.^1^ EC is mainly divided into esophageal adenocarcinoma and esophageal squamous cell carcinoma (ESCC); ESCC accounts for above 90% of EC in Asian countries. Radiotherapy (RT) is one of the most important treatments for EC, with a wide range of indications, including treatment of unresectable advanced stage EC, preoperative neoadjuvant therapy, adjuvant therapy, and primary treatment for patients unable to undergo surgery. Local control probability and survival have been improved with RT;^2^, ^3^ however, the response among patients varies. Although radiation eradicates a large proportion of tumor cells, some cells can survive. As a result, more than 50% of patients treated with RT relapse, have a 5‐year overall survival of 20%.^4^, ^5^ It is therefore reasonable to find new strategies to increase radiation sensitivity.

Traditionally, chemotherapeutic agents (e.g., 5‐fluorouracil, cisplatin, and paclitaxel) have major radiosensitizers; however, the toxicity to normal tissue has limited their clinical application. Recently, tumor‐specific targeting agents have been considered as promising approaches to improve radiation sensitivity without increasing adverse effects.^6^ The elaborate roles of multiple oncogenes and downstream signaling pathways in radiation biology have been elucidated in various tumor models. Understanding has increased significantly about some receptor tyrosine kinases (RTKs), such as epidermal growth factor receptor (EGFR), insulin‐like growth factor 1 receptor (IGF1R), fibroblast growth factor receptor (FGFR), and vascular endothelial growth factor receptor (VEGFR). The activation, expression, and intracellular translocation of RTKs are often the first events in the process of cancer, such as proliferation, migration, angiogenesis, immune evasion, and energetic adaptation, as well as resistance to radiation.^7^ Among RTKs, the effect of EGFR in RT has been extensively investigated. Aberrant activation of EGFR is involved in numerous radioresistance activities, such as decreased apoptosis, cycle redistribution, hypoxia, and increased DNA repair.^8^ It is worth noting that overexpression of EGFR is seen in more than 50% of ECs.^9^ Thus, targeting EGFR may be an attractive strategy to enhance radiation sensitivity in EC.

Cetuximab (IMC‐C225), a chimeric human–murine IgG1 monoclonal antibody, specifically targets the extracellular area of the EGFR to inhibit its activation. Clinical trial data have documented that cetuximab markedly sensitizes RT and improves outcomes in patients with non‐small cell lung cancer (NSCLC) and head and neck squamous cell carcinomas (HNSCC).^10^, ^11^, ^12^ Currently, cetuximab is approved by the FDA in combination with RT for advanced HNSCC. These successful trials encouraged radiation oncologists to investigate it in other types of tumor. However, trials with the addition of cetuximab in EC failed to show significant improvement in relapse and survival.^9^, ^13^ In addition, although patients were stratified in these trials according to tumor histological type, adenocarcinoma accounted for the majority. These results highlight the need to investigate whether ESCC responds to cetuximab and further identify potential populations that could benefit from cetuximab. Therefore, in order to optimize treatment, further study may be warranted to explore the special signal of cetuximab in radiotherapy sensitization of EC, especially its difference in other tumors such as head and neck squamous cell carcinoma. To our knowledge, there is still a lack of studies on the mechanism of cetuximab sensitization in EC (in particular, ESCC) to radiation.

To determine whether cetuximab improves radiation sensitivity in ESCC, we investigated the effect of cetuximab in radiation of ESCC cell lines ECA109 (high expression of EGFR) and TE‐13 (low expression of EGFR).^14^ Furthermore, cell proliferation, apoptosis, cycle distribution, DNA repair ability and molecular mechanisms were analyzed. Our results suggest an alternative strategy to enhance radiation sensitivity in ESCC.

## METHODS

### Cell lines and cell culture

The normal esophageal cell line HET‐1A (purchased from Shanghai Gaining Biotechnology Co., Ltd., Shanghai, China) and two ESCC cell lines ECA109 and TE‐13 (generously provided by the Fourth Hospital of Hebei Medical University, Hebei, People's Republic of China) were cultured in RPMI‐1640 medium supplemented with 10% fetal bovine serum (FBS) and penicillin (100 U/mL), and maintained in a humidified incubator with 5% CO2 at 37°C.^15^

### Reagents and irradiation

Cetuximab was obtained from clinical patient residues (Cancer Center, Union Hospital, Tongji Medical College, Huazhong University of Science and Technology, Wuhan, People's Republic of China) within 24 h of opening the packages and diluted to a certain concentration with RPMI‐1640 containing 1% FBS. Cells were irradiated using 6 MV photons generated by a medical linear accelerator (Primus K; Siemens) at room temperature. The transport dose rate was 1.2 Gy/min.

### MTT assay

The 3‐[4,5‐dimethylthiazol‐2‐yl]‐2,5 diphenyl tetrazolium bromide (MTT) assay is a measure of mitochondrial dehydrogenase activity within the cells and is thereby used for cell viability analysis. ECA109, TE‐13, and HET‐1A cells were plated in 96‐well plates at a density of 4000/well. The original culture solution was discarded 24 h later, and the cells were exposed to cetuximab at various concentrations (0, 1000, 500, 250, 125, 62.5, 31.25, 15.625, 7.8125, 3.90625 μg/mL) for 24, 48, and 72 h. Subsequently, 20 μL of MTT (5 mg/mL) with 200 μL of fresh medium was added into each well and incubated for 4 h. Then, the MTT solution was removed and the formazan crystals were solubilized in a solution containing 150 μL of dimethyl sulfoxide (DMSO). Cell viability was measured by a plate reader (PerkinElmer Singapore Pte. Ltd) at 490 nm. The cell inhibition rate was calculated as: [(Ac − At)/(Ac − Ab)] × 100%; where Ac = optical density (OD) of the control wells containing cells and MTT solution; At = OD of the treatment wells containing the cells, cetuximab, and MTT solution; and Ab = OD of control wells containing only MTT solution.

### Clonogenic survival assay

ECA109 and TE‐13 cells were plated in six‐well plates for 24 h under standard conditions, and then divided into two groups: radiation group, and cetuximab (500 μg/mL) + radiation group. The cells were pretreated with 1% FBS 1640 medium with or without 500 μg/mL cetuximab for 24 h at 37°C in a 5% CO_2_ atmosphere. Then, the radiation group and cetuximab + radiation group were irradiated at doses of 0, 2, 4, 6, and 8 Gy, and the solution was changed to 10% FBS 1640 medium. Then the cells were plated at low density and grown. After 14 days of cell culture under standard conditions, the colonies were fixed with methanol and stained with 0.5% crystal violet for 30 min. Colonies containing at least 50 cells were counted. The dose‐survival curve was fitted into the multitarget single‐hit model: survival fraction (SF) =1 − (1 − e‐D/D0)N with GraphPad Prism 7.0.0 (GraphPad Software). The radiobiological parameters including quasi‐threshold dose (Dq), mean lethal dose (D0), survival fraction at 2 Gy (SF2), and sensitivity enhancement ratio (SER) (SER = D0 control group/D0 combination group) were calculated.

### Flow cytometry analysis of cell cycle

ECA109 and TE‐13 cells were plated in six‐well plates for 24 h and divided into four groups: control group, cetuximab (500 μg/mL) group, radiation group, and cetuximab (500 μg/mL) + radiation group. The cells were exposed to 500 μg/mL cetuximab (cetuximab group and cetuximab + radiation group) for 24 h, and then irradiated at doses of 4 Gy (radiation group and cetuximab + radiation group). The solution was replaced with 10% FBS 1640 medium. After 24 h, cells were trypsinized, dissociated and fixed with precooled 70% ethanol overnight. Cells were washed with phosphate buffered saline (PBS) three times and incubated with 100 μg/mL RNAase for 30 min at 37°C. Then, propidium iodide (PI) was added to the cell suspension at a concentration of 50 μg/mL followed by incubation for 30 mi in darkness. The DNA content and cell cycle distribution were tested by the flow cytometer (FACScan; BD). The quantification of cells in different phases (G1, S, or G2/M) was analyzed using CELLQuest (BD).

### Flow cytometry analysis of cell apoptosis

As in the apoptosis assay, cells were collected 24 h after irradiation (4 Gy), washed three times with PBS and resuspended with 250 μL binding buffer. The resuspended cells were stained with 5 μL annexin V‐FTTC and 5 μL PI solution for 15 min at room temperature. The cells were then analyzed by flow cytometry.

### Immunofluorescence assay

Cells were plated in six‐well plates and divided into two groups: radiation group and cetuximab (500 μg/mL) + radiation group. Cultured cells were exposed to 2 Gy of radiation. Cells were washed in PBS three times and fixed in 4% paraformaldehyde at 0.5, 2, 6, and 24 h after irradiation. Then cells were permeabilized with 1% Triton X‐100 (in PBS) for 5 min and blocked with 2% bovine serum albumin (BSA) in PBS for 30 min at room temperature. The cells were stained with primary antibody (FITC‐conjugated anti‐phospho‐histone H2AX, Ser139) for 12 h at 4°C. After washing three times, the samples were incubated with the secondary antibody for 1 h at room temperature. After the same washing process, the cell nuclei were labeled with Hoechst dye for 10 min, and slides were mounted using 5% glycerol. The γH2AX foci were counted using a laser confocal microscope (Olympus Optical Co.).

### Western blot analysis assay

Cells pretreated with 500 μg/mL cetuximab for 24 h were irradiated at a dose of 2 Gy. After another 24 h, western blot analysis was performed. Cells were lysed with whole‐cell lysates and cell lysis buffer (radioimmunoprecipitation assay [RIPA] buffer and 1% phenylmethylsulfonyl fluoride [PMSF]). The proteins of cell extracts were collected and the concentrations of proteins were determined by a BCA kit (Applygen Technologies). Protein lysates were then separated into 8% or 10% sodium dodecyl sulfate–polyacrylamide gels according to the protein molecular weight and transferred to polyvinylidene difluoride (PVDF) membranes. Immunoblots were blocked with 5% BSA blocking buffer for 1 h at room temperature and probed with primary antibodies γ‐H2AX (97 148, Cell Signaling Technology), EGFR (2085, Cell Signaling Technology), phospho‐EGFR (p‐EGFR) (4407, Cell Signaling Technology), protein kinase B (AKT) (4691, Cell Signaling Technology), p‐AKT (4060, Cell Signaling Technology), extracellular regulated protein kinases (ERK) (4695, Cell Signaling Technology), p‐ERK (4370, Cell Signaling Technology), Ras (14 429, Cell Signaling Technology), RAD51 (8875, Cell Signaling Technology), and β‐actin (4970, Cell Signaling Technology) overnight at 4°C. After washing three times, the membranes were incubated with appropriate horseradish peroxidase‐labeled secondary antibody (D110058/D110098, Sangon Biotech) for 1 h at room temperature. We developed the membranes with the ECL AdvanceTM Western blotting detection kit to detect proteins on X‐ray film.

### Statistical analysis

Experiments were performed at least in triplicate. Data were presented as mean ± standard deviation (±SD). Student's *t*‐test and ANOVA were used to determine the statistical differences, and two‐sided *p* < 0.05 values were considered to be statistically significant.

## RESULTS

### Cetuximab alone was not sufficient to suppress cell viability

**FIGURE 1:**
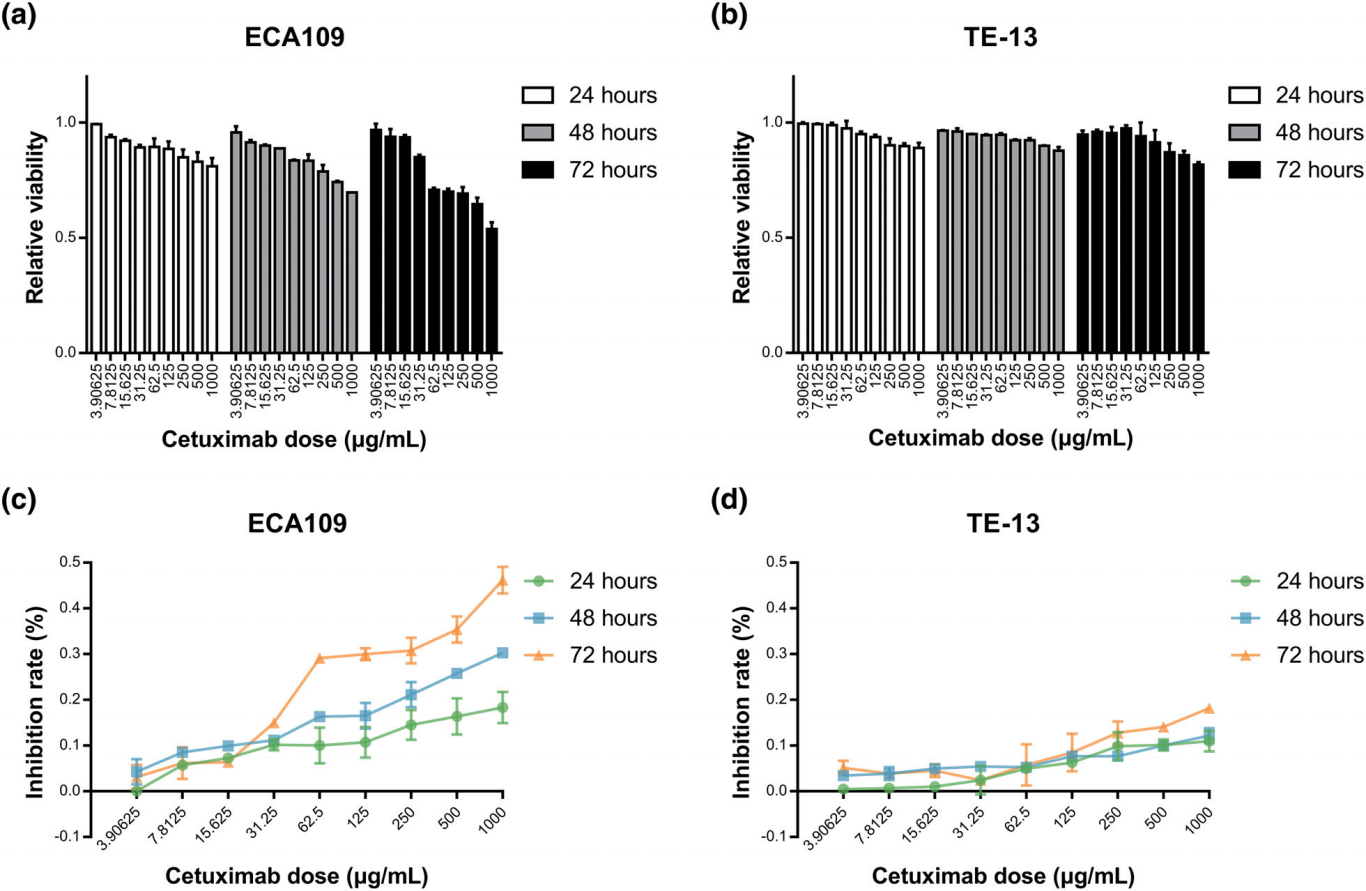
Cetuximab viability influence in ECA109 (a, c) and TE‐13 cells (b, d).

The antiproliferative effect of cetuximab at various concentrations on ECA109 and TE‐13 cells was evaluated using the MTT assay. Cell viability analysis was also performed on normal esophageal cell line HET‐1A, which is helpful for evaluating drug safety. HET‐1A viability remained almost unchanged, suggesting that cetuximab almost did not affect the activity of normal esophageal cells (File S1). The inhibition profiles of ECA109 and TE‐13 cell viability over 24, 48 and 72 h are shown in Figure 1. For both ECA109 and TE‐13 cells, the inhibition rate was not more than 20% over 24 h, 30% over 48 h, and 50% over 72 h. The inhibition rate was less than 50% even when cells were exposed to the highest dose level for 72 h incubation. The results indicated that cetuximab alone was not sufficient to suppress cell viability.

### Cetuximab enhanced the radiosensitivity of ECA109 and TE‐13 cells

**FIGURE 2:**
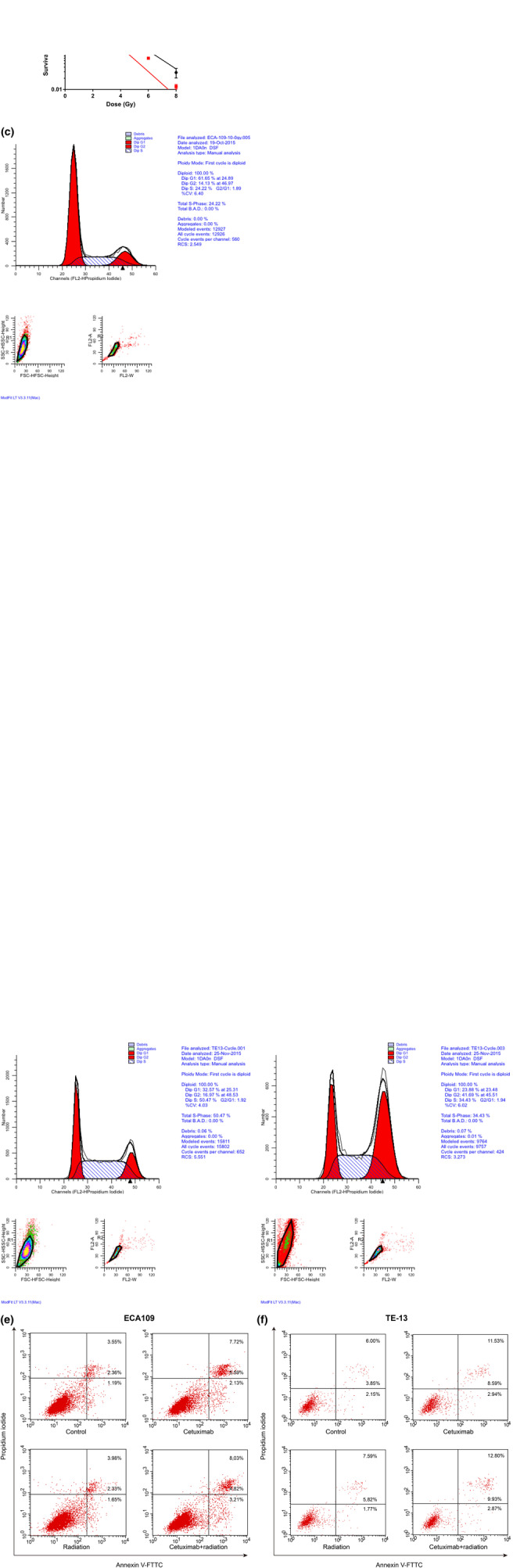
Cell clonogenic survival (a, b), cell cycle distribution (c, d) and cell apoptosis differences after irradiation with or without cetuximab in ECA109 (a, e, e) and TE‐13 cells (b, d, f). *p < 0.05, **p < 0.01, ***p < 0.001, ****p < 0.0001.

Clonogenic survival assays were performed to determine whether cetuximab was able to enhance radiosensitivity of ECA109 and TE‐13 cells with respect to cell proliferation. Cells were exposed to radiation (0, 2, 4, 6, and 8 Gy) after 24 h incubation with or without 500 μg/mL cetuximab. After 2 weeks of growth, 500 μg/mL cetuximab significantly decreased the number of colonies of irradiated ECA109 and TE‐13 cells, compared with untreated cells. As shown in Figure 2a,b and Table 1, dose‐survival curves revealed that cetuximab was associated with reduced SF in both ECA109 and TE‐13 cells with radiation from 2 to 8 Gy. The sensitivity enhancement ratio (SER) was 1.341 (in ECA109 cells) and 1.237 (in TE‐13 cells), suggesting that cetuximab was able to increase radiosensitivity of both ECA109 and TE‐13 cells.

**TABLE 1:** Radiation parameters fitted to multitarget single‐hit model.

Parameters	ECA109		TE13	
	Radiation	Cetuximab + radiation	Radiation	Cetuximab + radiation
Dq	2.261	1.504	2.081	1.345
D0	1.731	1.290	2.017	1.631
N	3.693	3.207	2.806	2.281
SF2	0.771	0.539	0.719	0.548
SER	1.341		1.237	

Abbreviations: D0, mean lethal dose; Dq, quasi‐threshold dose; N, extrapolation number; SER, sensitivity enhancement ratio; SF2, surviving fraction at 2 Gy.

### Cetuximab promoted G2/M cell cycle arrest of ECA109 and TE‐13 cells after radiation

To investigate the potential mechanisms by which cetuximab enhances radiosensitivity, we evaluated cell cycle progression by flow cytometry. As shown in Figure 2c,d and Table 2, cells exposed to radiation alone exhibited a mild increase in the percentage of the G2/M phase compared with that of controls. Cetuximab (500 μg/mL) alone was not sufficient to affect the cell cycle distribution compared with control cells. However, combination treatment of 500 μg/mL cetuximab and radiation induced a significant accumulation of cells in the G2/M phase (54.35 ± 2.87%) with a significant decrease in the G1 phase (40.52 ± 4.21%) in ECA109 cells compared with the radiotherapy condition (32.49 ± 3.89% of G2/M phase) or the control condition (9.44 ± 1.78% of G2/M phase) (both *p* < 0.05). Similar results were observed in TE‐13 cells (56.49 ± 0.21% in combination cells vs. 15.91 ± 0.87% in control cells' G2/M phase). Therefore, cetuximab increased radiation‐induced G2/M cell cycle arrest in EC cells.

**TABLE 2:** Cell cycle distribution in ECA109 and TE13 (^−^ *x* ± *s*).

	ECA109			TE13		
	G0/G1 (%)	G2/M (%)	S (%)	G0/G1 (%)	G2/M (%)	S (%)
Control	59.71 ± 3.38	9.44 ± 1.78	30.86 ± 1.60	33.84 ± 1.04	15.91 ± 0.87	50.26 ± 0.17
Cetuximab	58.51 ± 2.56	12.11 ± 1.65	29.39 ± 4.22	26.17 ± 1.08	16.28 ± 0.85	57.56 ± 1.93
Radiation	58.17 ± 4.92	32.49 ± 3.89	9.35 ± 1.02	19.67 ± 3.44	44.98 ± 2.69	35.36 ± 0.76
Cetuximab + radiation	40.52 ± 4.21	54.35 ± 2.87	5.14 ± 1.34	13.31 ± 1.03	56.49 ± 0.21	30.21 ± 1.25

We then conducted an apoptosis analysis by annexin V‐FITC and PI staining. The calculated total apoptosis rate is shown in Figure 2e,f. However, there was no significant difference in apoptotic cell percentage between the combination group and the irradiation group for both cell lines.

### Cetuximab enhances radiation‐induced DNA damage and delays DNA repair in ECA109 and TE‐13 cells

**FIGURE 3:**
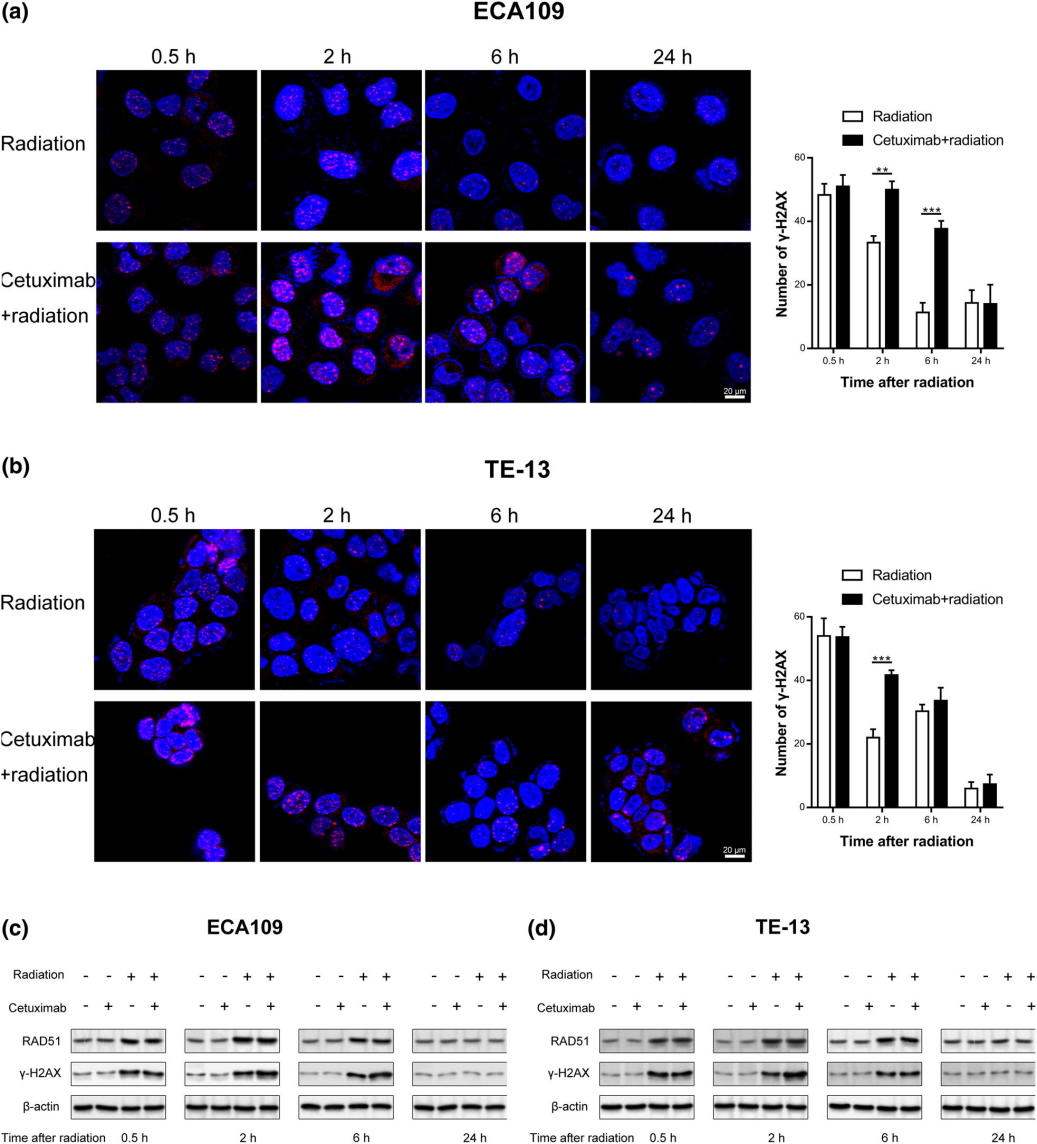
Illustration of γH2AX foci formation by immunofluorescence (a, b) and western blot assay (c, d). **p < 0.01, ***p < 0.001.

The major impact of radiation on cells is the induction of DNA DSBs and stimulation of DNA repair. The production of DSBs is accompanied by phosphorylation of the C‐terminal tail of the variant H2AX and γ‐H2AX foci formation at the DNA break sites.^16^ Therefore, persistence of γ‐H2AX foci is used as an indicator of lethal DNA damage. The immunofluorescence assay was performed to evaluate γ‐H2AX foci. Cells were exposed to 2 Gy of radiation, and kinetic changes of γ‐H2AX foci were examined at 0.5, 2, 6, and 24 h after irradiation. As shown in Figure 3a,b, the number of γ‐H2AX foci increased at 0.5 h after irradiation in radiation groups, and then continued to decrease. However, in ECA109 cells, the amount of γ‐H2AX foci at 2 and 6 h in the combination group was significantly higher than that in the radiotherapy alone group. In TE‐13 cells, a higher number of γ‐H2AX foci only at 2 h was observed in the combination group compared with the radiation group. These results indicated that cetuximab enhances radiation‐induced DNA damage and delays DNA repair in ECA109 and TE‐13 cells.

In addition, we validated the above results by western blot analysis assay. The level of total γ‐H2AX protein and RAD51 were observed after radiation exposure. The expression of γ‐H2AX protein was slightly increased in the combination treatment group compared with the radiotherapy alone group (Figure 3c,d). The expression of RAD51 was not significantly affected.

### Cetuximab reduced the activation of EGFR‐ERK signaling pathways in irradiated ECA109 and TE‐13 cells

**FIGURE 4:**
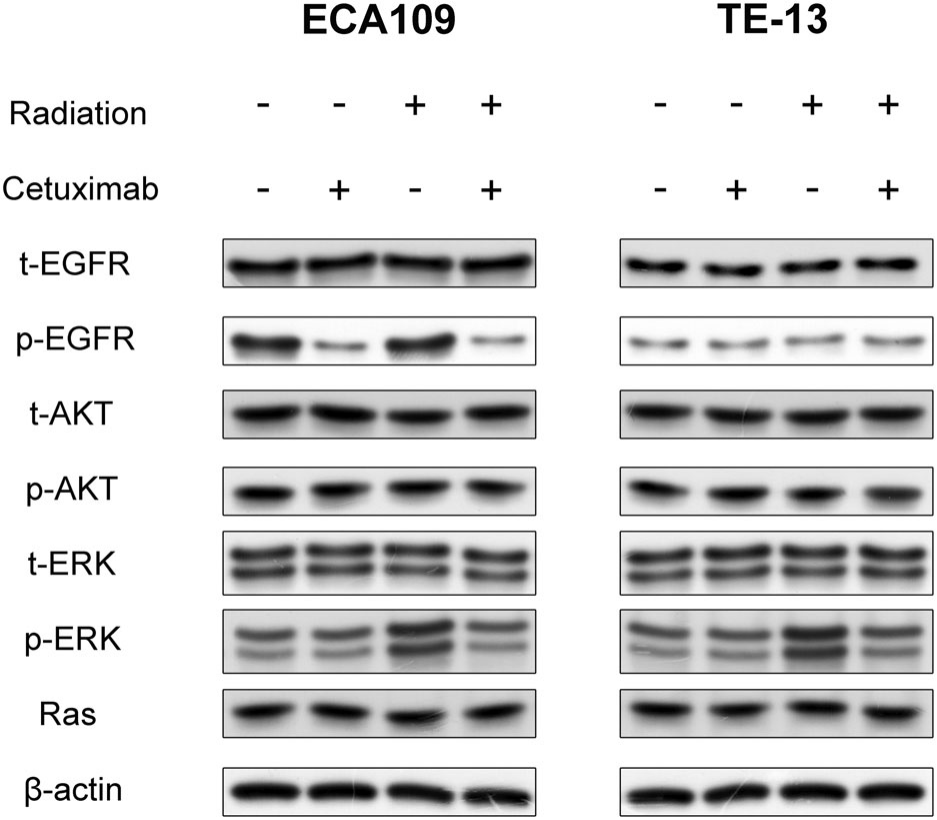
Expression differences of major proteins on the EGFR pathway after 24 h of 2 Gy irradiation with or without cetuximab in ECA109 and TE‐13 cells.

Downregulation of EGFR signaling pathways has been thought to play a major role in the effect of cetuximab. Therefore, several major proteins in the EGFR pathway were analyzed using western blot analysis assay. As shown in Figure 4, the expression of p‐EGFR was decreased in cells with cetuximab (cetuximab group or combination group), indicating that the involvement of EGFR in response to cetuximab (500 μg/mL) combined with radiation of ECA109 and TE‐13 cells. To further evaluate the underlying mechanism, we investigated the expression of total AKT, p‐AKT, total ERK, p‐ERK and Ras. As shown in Figure 4, the expression of p‐ERK decreased in the combination group compared with radiation alone group, that is, 500 μg/mL cetuximab inhibited the phosphorylation of ERK in irradiated cells. However, there were no significant differences in the levels of total EGFR, total AKT, p‐AKT, total ERK, and Ras among groups, suggesting that their levels remained stable even with the addition of cetuximab in irradiated cells.

## DISCUSSION

In this study, we found cetuximab was able to enhance the radiosensitivity of ESCC cells. With flow cytometry analysis, we demonstrated that cetuximab promoted G2/M cell cycle arrest in irradiated cells. By assessing γ‐H2AX foci formation with an immunofluorescence assay, we found that cetuximab increased radiation‐induced DNA damage and delayed DNA repair. These observations led us to investigate the role of signal pathways in the effects of cetuximab combined with radiation and found that cetuximab inhibited the phosphorylation of EGFR and ERK in irradiated cells.

The activation of EGFR and downstream signaling pathways, such as PI3K/AKT/mTOR and RAS/RAF/MEK/ERK, contributed to the resistance to radiation.^17^ EFGR inhibition has been an attractive treatment to improve radiosensitivity. Cetuximab is a classic EGFR antibody with universal clinical accessibility. The role of cetuximab in radiosensitivity in HNSCC and NSCLC has been widely studied with preclinical and clinical success. In this study, via the MTT assay and clonogenic survival assay, we demonstrated that cetuximab alone had few effects on ESCC cell viability but it significantly inhibited proliferation of irradiated cells. To our knowledge, there have been very few in vitro or in vivo studies of cetuximab combined with radiation in EC. Milas et al. studied nude mice bearing 8‐mm‐diameter A431 tumors treated with cetuximab and 18 Gy of single‐dose local tumor irradiation and found that cetuximab dramatically improved the efficacy of local tumor irradiation.^18^ In their study, histological analyses revealed that cetuximab caused heavy tumor infiltration with granulocytes, increased tumor cell terminal differentiation, and inhibited tumor angiogenesis when combined with radiotherapy. However, to clarify differences in the role of cetuximab in irradiated EC, we believe that it is more appropriate to investigate potential cellular and molecular mechanisms.

First, we evaluated cell cycle progression by flow cytometry. The results of our study suggested that combination treatment of cetuximab and radiation induced a significant accumulation of ESCC cells in the G2/M phase. It is well known that radiation sensitivity depends on the state of the cell cycle. The G2/M phase cells have the highest radiosensitivity, while S phase and G0 phase cells showed some resistance. Although in previous studies, EGFR inhibition always induced G1 arrest, which was associated with reduced activity of cyclin‐dependent kinase 2, cyclin A, and cyclin E;^19^ however, multiple studies still found that cetuximab promotes G2/M cycle arrest, especially under radiation, which is consistent with our conclusion.^20^, ^21^ The cell cycle is associated with a highly regulated set of events, in which cyclin‐dependent kinases, cyclins, checkpoints pathway, and regulatory factors are involved.^22^ Our study found that cetuximab lengthened the G2/M phase in irradiated cells, but further molecular investigation is needed. Regulation of the G2/M cell cycle maintains proper cell proliferation, and G2/M phase arrest often accelerates apoptosis. We observed a slight increase in the apoptotic rate of irradiated ESCC cells with cetuximab treatment, although there was no significant difference between groups. Our findings on apoptosis agree with those reported by Teng et al.^23^ and Kriegs et al.^24^ They demonstrated that EGFR inhibitors enhanced radiosensitivity through G2/M phase arrest but were not significantly associated with apoptosis.

We then investigated the role of DSB repair in cetuximab‐mediated radiosensitivity. Current preclinical studies show that EGFR modulates repair of DSBs via sharply increasing DNA‐dependent protein kinase catalytic subunits (DNA‐PKcs) and formation of the EGFR‐DNA‐PK complex.^25^, ^26^ Upon radiation, EGFR translocates to the nucleus and increases the activity of DNA‐PK.^26^ Homologous recombination (HR) and nonhomologous end joining (NHEJ) are the principal pathways responsible for the repair of radiation‐induced DSBs. NHEJ is the major repair mechanism in mammals that ligates broken DNA ends without the need for sequence homology. NHEJ is often initiated by Ku70/80, which has a high affinity for DNA ends. Once they are bound by Ku70/80, DNA‐PKcs are recruited, then DSBs are processed by Artemis, polymerase, ligase IV/XRCC4, XLF complex, and other proteins.^27^ Subsequently, the processed ends are ligated and DSBs are completely repaired. The γ‐H2AX foci have been widely accepted as a sensitive marker in repair of DSBs.^16^ We speculated that cetuximab‐mediated radiosensitivity is associated with EGFR blockade and subsequent reduction in activity of DSB repair. As we expected, the number of γ‐H2AX foci was reduced in the combination treatment group. This suggested that DSB repair (especially NHEJ) was delayed in irradiated ESCC cells treated with cetuximab. More importantly, this delay of DSB repair collaborates with G2/M arrest to enhance radiation‐induced cell death.^28^

We illustrated the regulation of EGFR and downstream pathways for cetuximab combined with radiation. Our data suggest that cetuximab significantly inhibited the phosphorylation of EGFR in irradiated ESCC cells. There are two major downstream signaling pathways, PI3K/AKT and RAS/RAF/MEK/ERK, so we further investigated the expression of biomarkers in these two pathways via western blot analysis assay. Based on our results, only decreased phosphorylation of EGFR and ERK was observed when cetuximab was combined with radiation, suggesting a potential role for the ERK pathway underlying the effect on ESCC radiosensitivity. The action of the ERK pathway is related to various molecular events. Previous studies suggested that EGFR signaling plays a proliferative role through RAS/RAF/MEK/ERK and an anti‐apoptotic role in radiation resistance through PI3K/AKT.^17^, ^29^ These studies are consistent with our results. Our data demonstrated that cetuximab inhibited ERK signaling and mediated G2/M arrest in irradiated cells; however, it was not significantly associated with apoptosis. The ERK signaling is also involved in radiation‐induced formation of γ‐H2AX foci.^30^, ^31^ In addition, the phosphorylation level of AKT exhibited no significant changes between groups, suggesting that the AKT pathway was not significantly affected by cetuximab combined with radiation. By comparing the p‐ERK level with p‐AKT, our results revealed that inhibition of the ERK pathway plays a more crucial role in cetuximab‐mediated radiosensitivity of ESCC than dose the AKT pathway. This is an interesting phenomenon, in view of the difference in head and neck cancer and lung cancer. Many in vivo and in vitro studies have suggested that the mechanism of radiotherapy sensitization by cetuximab in HNSCC or NSCLC is likely due to the inhibition of the PI3K/AKT pathway.^32^

Recently an increasing number of clinical trials have evaluated the combination of multiple targeted therapies, and satisfactory toxicity and efficacy have been achieved.^33^, ^34^ The rationale is based on the potentially complementary mechanisms of the drug and radiotherapy combination to increase the response, minimize overlapping toxicities and overcome resistance. For HNSCC, preclinical data have suggested that dual inhibition of EGFR and the PI3K/AKT pathway plays a synergistic role in proliferation suppression and radiosensitivity enhancement.^35^, ^36^ Consequently, related clinical trials of combination treatment have been conducted. Dunn et al. investigated the addition of BYL719, a PI3K/AKT inhibitor, to cetuximab and radiation in advanced HNSCC.^33^ Although their study was only a Phase Ib clinical trial and thus did not provide enough evidence for efficacy, their multiple‐targeted therapy provides a novel strategy for improving radiosensitivity. For ESCC, our study demonstrated that ERK is a potential downstream pathway for cetuximab in radiosensitivity. Therefore, we speculated that the addition of ERK pathway inhibitors would further enhance the efficacy of cetuximab, which may have implications for future clinical trials of radiotherapy sensitization in ESCC. In addition, technological advancements over recent years mean that nanomedicine has enormous potential in drug delivery, drug absorption, and therapeutic efficiency.^37^, ^38^

Clearly, the molecular investigation of this study is from a limited perspective. Further studies of a more detailed relationship between cetuximab and radiation are needed to understand the mechanism and translate it into clinical practice.

In conclusion, this study revealed that cetuximab improved radiosensitivity in ECA109 and TE‐13 cells. It may act by promoting G2/M cycle arrest and reducing DSB repair, as well as inhibiting EGFR and downstream ERK pathways. Our findings may provide a potential therapeutic strategy as an adjuvant in radiotherapy of ESCC.

## AUTHOR CONTRIBUTIONS

Guifang Zhao undertook the experiments and analysed the data. Liwen Feng drafted the first draft of the manuscript, and aided in the experiments. Ting Ye, Yanshen Liu, Li Fan helped manuscript editing. Jing Chen and Chengzhi Ye conceived and designed the study. All authors reviewed and critiqued the manuscript, and agreed to the final submission of the manuscript.

## CONFLICT OF INTEREST STATEMENT

All authors declared no conflicts of interest in this manuscript.

## Supporting information


**File S1.** Influence of cetuximab on relative viability (A) and inhibitory rate (B) of HET‐1A.
