# Supplementary figures and images for: Cetuximab enhances radiosensitivity of esophageal squamous cell carcinoma cells by G2/M cycle arrest and DNA repair delay through inhibiting p‐EGFR and p‐ERK
Source: Thorac Cancer. 2023 Jun 20;14(22):2127–38. doi: 10.1111/1759-7714.14995 (PMC10396788; doi:10.1111/1759-7714.14995)

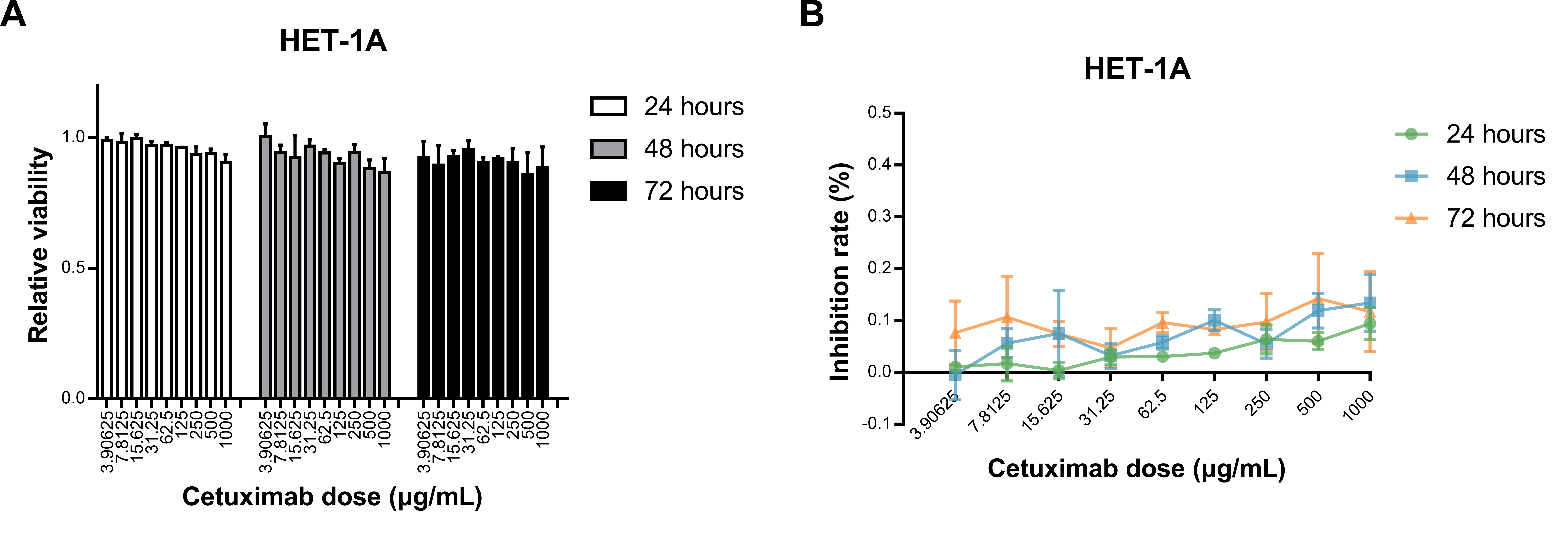

Supplement: Supplementary file 1 — File S1. Influence of cetuximab on relative viability (A) and inhibitory rate (B) of HET‐1A. [file TCA-14-2127-s001.tif]
